# Supplementary material for: Tissue distribution of Coxiella burnetii and antibody responses in macropods co-grazing with livestock in Queensland, Australia
Source: PLoS One. 2024 May 21;19(5):e0303877. doi: 10.1371/journal.pone.0303877 (PMC11108133; doi:10.1371/journal.pone.0303877)
Supplement: S1 Table — (DOCX) [file pone.0303877.s001.docx]

**S1 Table. Primers used in real-time PCR reactions in this study.**

| **Gene** | **Primer/ probe** | **Sequence (5’ – 3’)** | **Amplicon size (bp)** | **Final conc. (nM)** | **Reference** |
| --- | --- | --- | --- | --- | --- |
| IS*1111* | IS1111-F | AAAACGGATAAAAAGAGTCTGTGGTT | 70 | 300 | [39] |
|  | IS1111-R | CCACACAAGCGCGATTCAT |  | 300 |  |
|  | IS1111-P | Quasar670^a^-AAAGCACTCATTGAGCGCCGCG-BHQ2^b^ |  | 150 |  |
| *com1* | com1-F | AAAACCTCCGCGTTGTCTTCA | 76 | 400 | [40] |
|  | com1-R | GCTAATGATACTTTGGCAGCGTATTG |  | 400 |  |
|  | com1-P | FAM^c^-AGAACTGCCCATTTTTGGCGGCCA-BHQ1^d^ |  | 200 |  |
| *htpAB* | htpAB-F | GTGGCTTCGCGTACATCAGA | 114 | 400 | [41] |
|  | htpAB-R | CATGGGGTTCATTCCAGCA |  | 400 |  |
|  | htpAB-P | FAM^c^ -AGCCAGTACGGTCGCTGTTGTGGT -BHQ1^d^ |  | 200 |  |
| *lin02483* | lipHQ-F | AACCGGGCCGCTTATGA | 62 | 50 | [43] |
|  | lipHQ-R | CGAACGCAATTGGTCACG |  | 50 |  |
|  | lipHQ-P | HEX-TTCGAATTGCTAGCGGCACACCAGT -BHQ1^d^ |  | 100 |  |

^a^ Quasar 670 carboxylic acid

^b^ Black Hole Quencher-2

^c^ 6-Carboxyfluorescein

^d^ Black Hole Quencher-1
