# Supplementary material for: Tissue distribution of Coxiella burnetii and antibody responses in macropods co-grazing with livestock in Queensland, Australia
Source: PLoS One. 2024 May 21;19(5):e0303877. doi: 10.1371/journal.pone.0303877 (PMC11108133; doi:10.1371/journal.pone.0303877)
Supplement: S2 Table — (DOCX) [file pone.0303877.s002.docx]

**S2 Table. The number of kangaroos and distribution of tissues that were positive for C. burnetii on the initial screening PCR for IS1111.**

| **Tissue - sample type** | **Total tested** | $\boldsymbol{n}$ **pos** | **% pos (95% CI)** |
| --- | --- | --- | --- |
| Heart | 50 | 25 | 50 (35, 64) |
| Lung | 50 | 17 | 34 (21, 49) |
| Mediastinal lymph node | 44 | 12 | 27 (15, 43) |
| Mesenteric lymph node | 50 | 19 | 38 (25, 53) |
| Spleen | 50 | 14 | 28 (16, 42) |
| Liver | 50 | 13 | 26 (15, 40) |
| Testes | 13 | 3 | 23 (5.0, 54) |
| Epididymis | 12 | 4 | 33 (9.9, 65) |
| Uterus | 35 | 13 | 37 (21, 55) |
| Pouch | 36 | 14 | 39 (23, 56) |
| Urine/bladder ^a^ | 48 | 20 | 42 (28, 57) |
| Faeces | 50 | 14 | 28 (16, 42) |
| Serum | 49 | 11 | 22 (12, 37) |
| *Total kangaroos* | *50* | *50* | *100 (93, 100)* |

CI: confidence interval.

^a^ Three urine samples, 45 bladder wall samples.
